# Autocrine Extra-Pancreatic Trypsin 3 Secretion Promotes Cell Proliferation and Survival in Esophageal Adenocarcinoma

**DOI:** 10.1371/journal.pone.0076667

**Published:** 2013-10-11

**Authors:** Song Han, Constance W. Lee, Jose G. Trevino, Steven J. Hughes, George A. Sarosi

**Affiliations:** 1 Department of Surgery, University of Florida College of Medicine, Gainesville, Florida, United States of America; 2 North Florida/South Georgia VA Medical Center, Gainesville, Florida, United States of America; Wayne State University, United States of America

## Abstract

Trypsin or Tumor associated trypsin (TAT) activation of Protease-activated receptor 2 (PAR-2) promotes tumor cell proliferation in gastrointestinal cancers. The role of the trypsin/PAR-2 network in esophageal adenocarcinoma (EA) development has not yet been investigated. The aim of this study is to investigate the role of trypsin/PAR-2 activation in EA tumorogenesis and therapy. We found that esophageal adenocarcinoma cells (EACs) and Barrett’s Metaplasia (BART) expressed high levels of type 3 extra-pancreatic trypsinogen (PRSS3), a novel type of TAT. Activity of secreted trypsin was detected in cultured media from EA OE19 and OE33 cultures but not from BART culture. Surface PAR-2 expression in BART and EACs was confirmed by both flow cytometry and immunofluorescence. Trypsin induced cell proliferation (∼ 2 fold; P<0.01) in all tested cell lines at a concentration of 10 nM. Inhibition of PAR-2 activity in EACs via the PAR-2 antagonist ENMD (500 µM), anti-PAR2 antibody SAM-11 (2 µg/ml), or siRNA PAR-2 knockdown, reduced cell proliferation and increased apoptosis by up to 4 fold (P<0.01). Trypsin stimulation led to phosphorylation of ERK1/2, suggesting involvement of MAPK pathway in PAR-2 signal transduction. Inhibition of PAR-2 activation or siRNA PAR-2 knockdown in EACs prior to treatment with 5 FU reduced cell viability of EACs by an additional 30% (P<0.01) compared to chemotherapy alone. Our data suggest that extra-pancreatic trypsinogen 3 is produced by EACs and activates PAR-2 in an autocrine manner. PAR-2 activation increases cancer cell proliferation, and promotes cancer cell survival. Targeting the trypsin activated PAR-2 pathway in conjunction with current chemotherapeutic agents may be a viable therapeutic strategy in EA.

## Introduction

Barrett’s esophagus (BE) is a condition characterized by the development of intestinal metaplasia of the esophageal mucosa. The clinical importance of this relatively common condition relates to its role as a precursor lesion to esophageal adenocarcinoma (EAC), entailing a 100-fold increased risk of developing EAC [1]. BE is associated with chronic gastroesophageal reflux disease (GERD), a chronic regurgitation of gastric fluid into the lower esophagus [2]. The gastric refluxate contains gastric secretions (acid and pepsin) as well as biliary and pancreatic secretions (bile salts and trypsin). The cellular and molecular mechanisms underlying the development Barrett’s esophagus and its progression to cancer remain unclear. Our previous work showed that bile salt glycochenodeoxycholic acid (GCDA) activates ERK/MAPK pathway to produce a pro-proliferative effect in a Barrett’s cell line [3]. However, it is unclear whether trypsin in refluxate also contributes to promote cell proliferation in these metaplastic cells.

The classic notion of trypsin playing a role in tumor invasion and metastasis due to proteolytic degradation of extracellular matrix (ECM) proteins has been challenged. Recent studies have revealed that the pro-tumorigenic role of trypsin could also be attributed to its function as a potent activator for G protein-coupled receptors; in particular, protein activated receptor 2 (PAR-2) [4]–[6]. Trypsin cleaves and activates PAR-2 more efficiently than any other PAR members (PAR-1, PAR-3 and PAR-4) [7], [8]. Cleaved by trypsin, PAR-2 exposes a new amino terminus peptide that functions as tethered ligand; this new ligand then binds to the core of the receptor itself and initiates signal transduction. Darmoul and colleagues demonstrated that tryspin serves as a very robust growth factor for colon cancer cell HT29 via activation of PAR-2 and downstream ERK phosphorylation [9]. In like manner, trypsin regulation of cellular adhesion and proliferation mediated by PAR-2/G-protein signaling has been reported in other malignancies such as breast cancer and gastric cancer [6], [10], [11].

Despite increasing evidence of trypsin induced activation of PAR-2 in cancer progression in other neoplasms, including digestive tract tumors such as gastric and colonic cancers, the functional consequences of trypsin evoked PAR-2 activation in esophageal cancer has not yet been reported. In this study, we hypothesized that the trypsin/PAR-2 axis may play a role in neoplastic progression in esophageal adenocarcinoma. We investigated the expression of PAR-2 and trypsin/trypsinogen in human immortalized Barrett’s cell line (BART) and human esophageal adenocacinoma cell lines OE19, OE33 and FLO1, and examined the effect of trypsin activated PAR-2 on cell proliferation and survival in these cell lines. We have also shown that inhibition of PAR-2 by various approaches sensitizes EAC cells to cytotoxic agents. Our results suggest that potent PAR-2 inhibitors could be new auxiliary therapeutic agents for esophageal cancer.

## Materials and Methods

### Cells Culture and Treatment

Three esophageal adenocarcinoma cell lines were chosen for this study based on the suggestion of Boonstra, *et al.* [12]. OE19 and OE33 were obtained from American Type Culture Collection (Rockville, MD). FLO1 has been previously described [13]. The human Barrett’s-derived non-neoplastic cells (BART) were telomerase-immortalized, a kind gift from Dr. Souza [14], OE33, OE19 and FLO1 were routinely cultured in 10 cm dishes and maintained in CO_2_ incubator in RPMI-1640 medium supplemented with 10%FBS and 1% penicillin-streptomycin. BART cells were cultured as previously described [3]. The optimal final concentrations for treatments are: 10 nM trypsin; 10 µg/ml leupeptin hemisulfate (Sigma-Aldrich, St Louis, MO); 500 µM ENMD (Enzo, Farmingdale, NY); 2 µg/ml Neutralizing PAR-2 antibody SAM-11 (Santa Cruz, Santa Cruz, CA).

### Quantitative Real-time PCR (q-RT-PCR) and cDNA Sequencing

Total RNA was extracted from cultured cells using TRIzol reagent (Invitrogen, Grand Island, NY) according to the manufacturer’s instructions. Reverse transcription was conducted for 60 min at 42°C from purified total RNA using iScript cDNA synthesis kit (BioRad, Hercules, CA). cDNA samples were subjected for q-RT-PCR performed on ABI 7500 FAST PCR system using SYBR Green PCR Kit (Qiagen, Valencia, CA). The housekeeping gene β_2_-miccroglobulin (β_2_M) was employed for normalization. Primers designed for the respective genes are listed in Table 1.

**Table 1:** Primer sequences for Real Time PCR.

Gene name	Gene ID	Forward Primer	Reverse Primer
Trypsinogen1	TRY1, PRSS1	CCACCCCCAATACGACAGGAAG	GCGCCAGAGCTCGCAGT
Trypsinogen2	TRY2, PRSS2	CCAAATACAACAGCCGG	AGTCGGCACCAGAACTCAGA
Trypsinogen3	TRY3, PRSS3	ACCCTAAATACAACAGGGAC	AGCACCAAAGCTCAGAGT
Pan-Trypsinogen		CGAGCTGCAGTGCCTGGATG	TCTTTCCAGGGTAGGAGGCTT
Protease-activated receptor 1	PAR 1, F2R	GCTGGTGGCCGCCTGCTTCAG	ACCGGGGATCTAAGGTGGCATTTGT
Protease-activated receptor 2	PAR 2, F2RL1	CCGATTCGGGGCAGGTGAGAGGC	CTCCGCATCCTCCTGGAAGCCCC
Protease-activated receptor 3	PAR 3, F2RL2	TGCCTGCACGGCACAGGAGA	TGGGGGAGCTCCACGAAAGGTC
Protease-activated receptor 4	PAR 4, F2RL3	AGCGCCTGGGGCAACCTCTA	CGTTGGAAGAGCCCTGCCCG

For DNA sequencing analysis, PCR amplicons were generated based on the primer pair homogeneous to all three types of trypsinogen and flanking type specific mismatches (Forward: CGAGCTGCAGTGCCTGGATG; Reverse: GTAGACCTTGGTGTAGACTCCAGGC). The purified PCR pan-trypsinogen amplicons were sent to the Interdisciplinary Center for Biotechnology Research in University of Florida for DNA sequencing analysis.

### PAR-2 Surface Expression Analysis

Cell surface PAR-2 expression was determined with immunofluorescent staining (IFC) and flow cytometry analysis. For IFC, cells were cultured on AF (Cascade Biologics) pre-coated glass bottom microwell dishes. At 60% confluence, cells were fixed in 4% paraformaldehyde and after three washes, incubated with anti-PAR-2 antibody N-19 (Santa Cruz) at 1∶50 diluted in 5% BSA/5% donkey serum/PBS at room temperature for 2 hour. The primary binding antibody was detected with Alexa Fluor 594 conjugated donkey anti-goat IgG (Invitrogen) at 1∶1000 dilution. Imaging was performed using an Olympus IX81-DSU Spinning Disk confocal microscope.

For flow cytometry analysis, cultured cells were detached gently by TrypLE™ Select (Invitrogen) and collected in PBS/0.5%BSA, and freshly stained with PE labeled anti-human PAR-2 antibody (R&D system) according to the manufacturer’s instructions. Labeled cells were analyzed using an LSRII flow cytometer (BD Biosciences).

### Immunoblot Analysis

Protein samples were collected in cold lysis buffer (Cell Signalling). 30 µg of total protein were separated by size fraction with 10% Mini-Protein TGX precasted gel (BioRad, Hercules, CA), transferred onto PVDF membranes, blocked with 5% milk protein, and incubated overnight with primary antibodies at 4°C. Blots were then incubated with HRP-conjugated secondary antibodies (Sigma-Aldrich) for 1 h at room temperature, and developed with the chemi-luminescence system (Pierce Biotechnology). Densitometric analysis was carried out using the ImageJ software (National Institutes of Health). Bands were boxed and background signal was subtracted from their relative intensities.

### Cell Proliferation, Cell Viability and Apoptosis Assay

Cell proliferation was determined by the production of ATP using ATP level kit (ViaLight Plus Kit, Lonza). Treated cells were extracted with lysis buffer supplied with the kit. Total ATP level in cells was measured by the luminescent readings in a luminometer (PolarStar Omega, BGM). Background of reagents-only reading was subtracted before data analysis. Each test was carried out at a minimum of six replicates. Luminescence readings outside of range of mean±2SD were excluded for analysis. MTT assay was employed in the assessment of cell viability. 10 K cells were seeded into each well of 96-well plates. On the day of testing, 20 µl of MTT was added into each well and the reaction allowed proceeding for 4 hrs at 37°C. 200 µl of DMSO was added to each well to dissolve the enzymatic product formazan and absorbance at 570 nm was measured in a plate reader (PolarStar Omega). To study for apoptosis, both floating and attached cells were pooled and analyzed using Annexin V/7-ADD staining (Annexin V/7-ADD Apoptosis Detection Kit I, BD Biosciences) by flow cytometry on a BD Biosciences LSRII.

### Trypsin Activity Assay

All cells were plated into 6-well plates at a density of 500 K cells per well for 24 hrs. Aliquots of cultured medium were collected at time points of 5, 15, 30, 60 min and 24, 48 hrs after changing into serum-free medium. Trypsin activity was monitored by the amount of released *p*-nitroanilide (*p*NA) from a specific substrate, measuring spectrophotometric units at 405 nm (A_405_) (Trypsin Activity Assay Kit, BioVision). Standard curve was built on two-fold serial diluted concentrations of the substrate *p*-NA from 20 to 2 nmol/well. 10 nM trypsin was used as positive control. Trypsin activity was calculated according to the formula suggested in the manufactory’s instruction.

### siRNA Transfection

The siRNA library contains On-TargetPlus SMART pools (L-00595, Dharmacon) of four siRNA duplexes targeting human F2RL1 gene (PAR-2, NM_005242). Negative control siRNA was an On-TargetPlus non-targeting pool (D-001810-10-05, Dharmacon). Sequences of the individual duplexes are GGUAUUGGGUCAUCGUGAA (J-005095-07), GUUAAGACCUCCUAUUGAG (J-005095-08), CUAGUAACCUUCUGCUUGU (J-005095-09) and GUGGUGGGUUUGCCAAGUA (J-005095-10). Cells were transfected with 25 nM siRNA using Lipofectamine RNAiMAX reagent (Invitrogen) according to the manufacturer’s protocols.

### Statistical Methods

All statistical analyses were performed using GraphPad Prism 5 software. Two-way analysis of variance ANOVA was used for pairwise comparisons between test groups. Data points are reported as experimental averages and error bars represent S.D. Results were deemed statistically significant at the level of *P*<0.05.

## Results

### Constitutive PAR-2 Surface Expression in BART Cells and EACs

**Figure 1:**
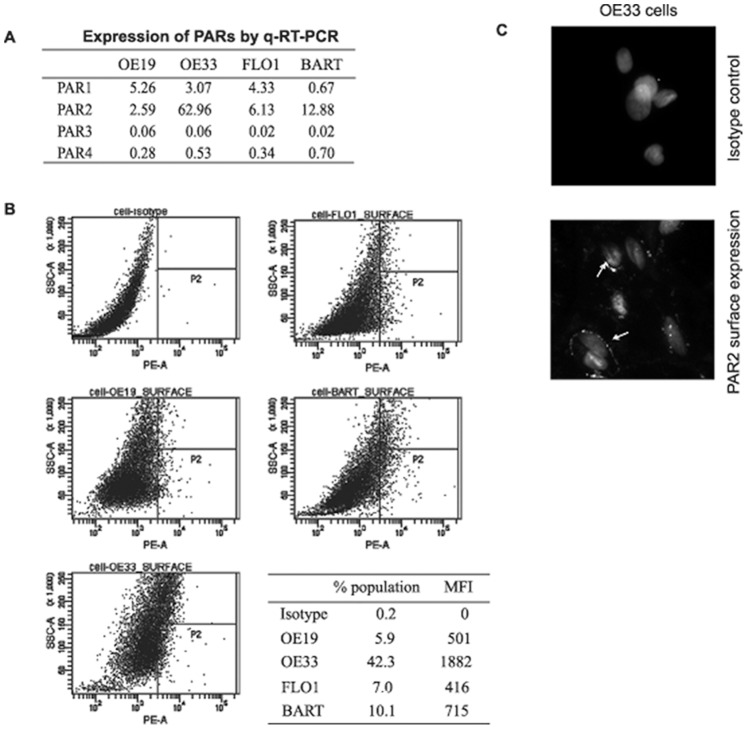
Cell immnunofluorescent staining for determination of PAR-2 surface expression. (A) RNA expression profiling of PAR members by q-RT-PCR. (B) Contour plot of SSC vs. anti-PAR-2 antibody PE fluorescence. All cells subjected to analysis were first plotted by FSC (forward light scatter) vs. SSC (side light scatter) and gated to exclude debris and clumps. P2 is set for gating positive cells based on isotype control. Affiliated table shows percentage of positive cells (% of population) and MFI (mean fluorescent intensity, value subtracted from isotype background). (C) Representative image of immunofluorescence staining of PAR-2 on OE33 cell surface. Top picture shows the isotype control staining. Arrows in bottom picture indicate cell membrane staining for PAR-2 with anti-PAR-2 antibody N-19. Cell nuclei were stained with DAPI. Offset and gain values of the photomultiplier channel were regulated with respect to the setup selected for isotype negative control to make fluorescence intensity comparable across all samples.

The PAR family consists of four individual members, PAR-1, PAR-2, PAR-3 and PAR-4. To investigate the PAR transcription profile in BART cells and EACs, a quantitative real-time RT-PCR (q-RT-PCR) was employed. With primers specific to each subtype of PAR genes (see Table 1), quantitative RT-PCR reveals the transcription of PAR-1 and PAR-2 (figure 1A) in all cells, while PAR-3 and PAR-4 transcription was found at a comparatively lower level. Among all cells, PAR-2 surface expression was found at high levels in both the premalignant BART cell line as well as in Barrett’s-derived adenocarcinoma cell line OE33. Flow cytometric analysis and immunocytofluorescence (ICF) detected PAR-2 protein surface expression in all cell lines tested (figure 1B and 1C).

### Trypsin Stimulates Cell Proliferation in BE Premalignant Conditions and EAC

**Figure 2:**
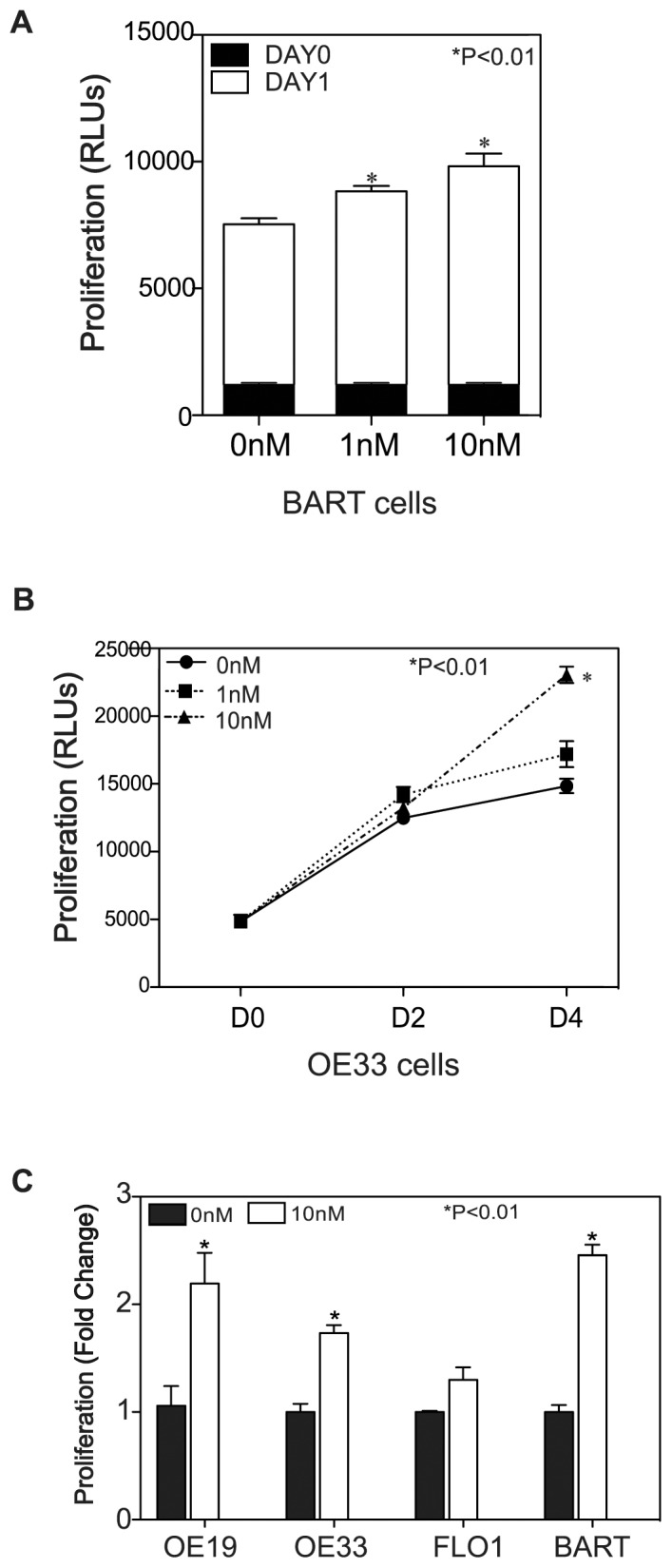
Proliferative effect of trypsin on cell proliferation. (A) BART cells were exposed to trypsin containing or control media. Day 1 after trypsin stimulation both 1 nM and 10 nM showed an increased ATP levels comparing to untreated cells (p<0.01). (B) Showing that trypsin induced OE33 cell proliferation at dose-dependent and time-dependent manner. OE33 cells were exposed to trypsin and left in serum-free medium for up to four days in the presence or absence of trypsin (1 nM or 10 nM). ATP levels in cell lysates were measured by Vialight® Plus Kit on day 0, day 2 and day 4. Stimulation with 10 nM trypsin showed a significant increase of ATP level comparing to untreated cells at day 4 post-treatment (p<0.01). (C) Depicting that 10 nM trypsin successfully stimulated cell growth in OE19, OE33 and BART cells in comparing with control groups, but failed to induce proliferation in FLO1 cells. Cell proliferation expressed as fold changes based on increase of arbitrary units of ATP chemiluninescent reading compared to untreated control group.

Barrett’s esophagus (BE) is a premalignant condition that predisposes to the development of esophageal adenocarcinoma. A progressive increase in cell proliferation has been suggested as one of the key cellular events underlying malignant transformation. We thus investigated whether trypsin could act as a growth factor in BART cells, stimulating cell proliferation. Cell proliferation was assessed by measuring cellular ATP levels using ViaLight® Plus Kit. BART cells were exposed to trypsin at concentrations of 1 nM or 10 nM. At each concentration, trypsin produced a statistically significant (p<0.01) proliferation increase over control. Although BART cells responded to trypsin at the lower concentration of 1 nM, 10 nM trypsin induced more potent response (∼2-fold, p<0.01) compared to control group (figure 2A). We then examined this pro-proliferative function of trypsin in the three EA cell lines OE19, OE33 and FLO1. Unlike BART cells, OE19 and OE33 cell did not significantly respond to 1 nM trypsin stimulation (p>0.05), yet 10 nM trypsin was able to potently promote cell proliferation (OE33 cell dose response over a 4-day time course shown in figure 2B). In FLO1 cells trypsin at both concentrations did not promote significant cell proliferation. Given this finding, we selected 10 nM trypsin as our model treatment for the remainder of our experiments. Figure 2C showed that 10 nM trypsin significantly induced proliferation in OE19 and OE33 over a 4-days time course and BART cells over a 1-day time course [an average fold-increase of ATP levels of 2.19±0.29, 1.73±0.07, and 2.46±0.12 respectively (p<0.01)], while FLO1 did not exhibit a significant response to 10 nM trypsin stimulation.

### ERK/MAPK Activation Mediates Trypsin Induced Proliferation

**Figure 3:**
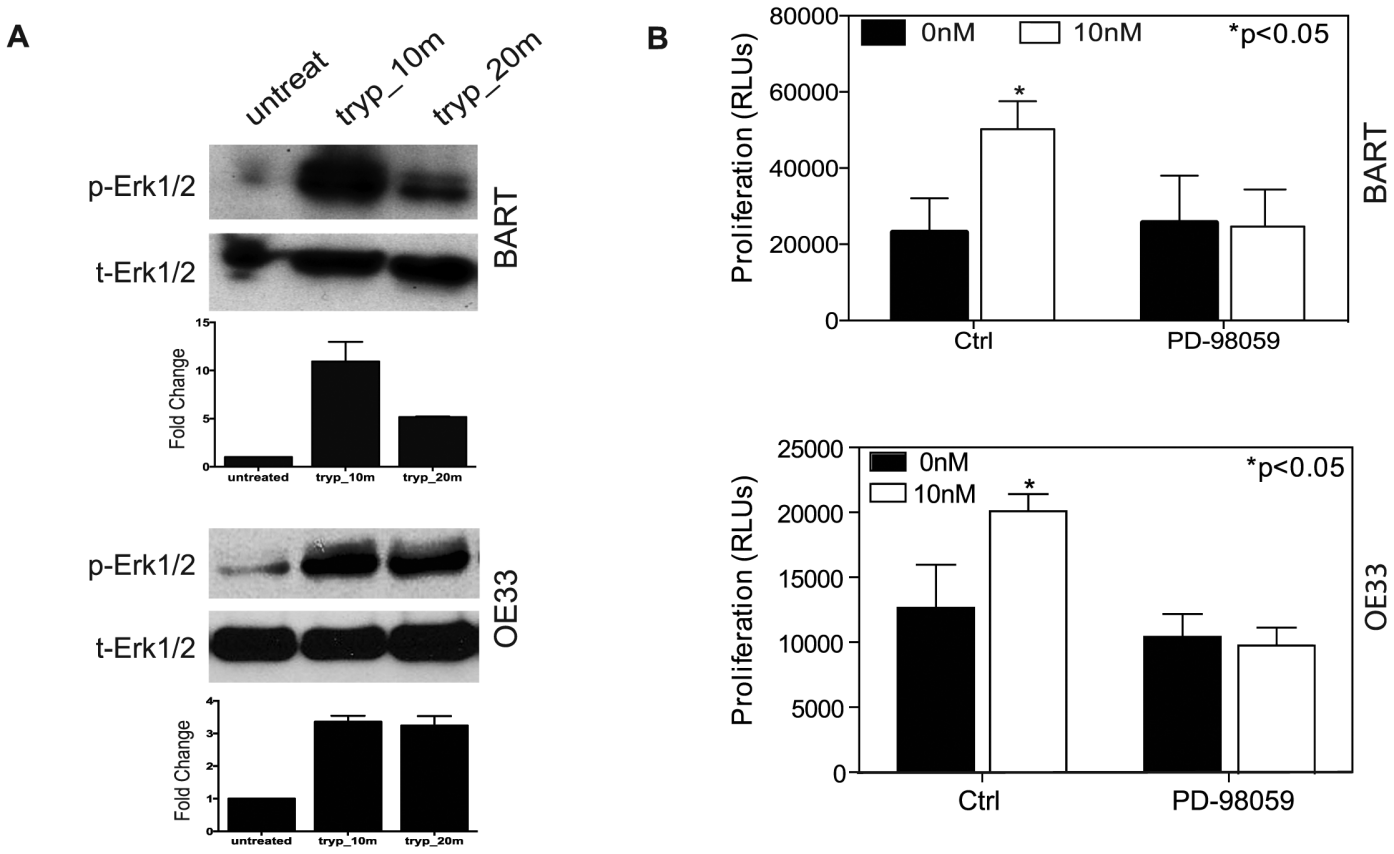
Erk/MAPK involvement in Trypsin Induced Signaling. (A) Showing representative photographs for Western blot analysis of Erk1/2 phosphorylation status in BART and OE33 cells in the presence of or the absence of 10 nM trypsin at different time course as indicated. (B) Proliferation assay revealed that MEK inhibition abolished the proliferative responses of both BART and OE33 cells to 10 nM trypsin stimulation.

The ERK/MAPK pathway plays a pivotal role in regulating cell proliferation. We thus investigated whether MAPK pathway was involved in trypsin/PAR-2 activation induced cell proliferation. BART and OE33 cells were exposed to 10 nM trypsin at time courses of 10 min and 20 min. By western blot analysis, in both BART cells and OE33 cells, 10 nM trypsin significantly increased phosphorylation of Erk1/2 after 10 min treatment (figure 3A). Intriguingly, prolonged effects on Erk1/2 phosphorylation upon trypsin stimulation were dissimilar between BART and OE33 cells. At 20 min after trypsin stimulation, BART cells showed a regression of Erk1/2 phosphorylation status, while in OE33 cells, extending trypsin stimulation to 20 min caused the increased Erk1/2 phosphorylation levels to persist (figure 3A). To confirm that trypsin-promoted cell proliferation was MAPK dependent, BART and OE33 cells were pretreated with MEK inhibitor PD-98059 at 20 µM prior to trypsin stimulation. As shown in figure 3B, the MEK inhibitor PD-98059 abolished the accelerated proliferation by trypsin/PAR-2 activation in both BART and OE33 cells; providing supportive evidence that trypsin stimulated cell proliferation was MAPK dependent.

### Trypsin Induced Proliferation is PAR-2 Dependent

**Figure 4:**
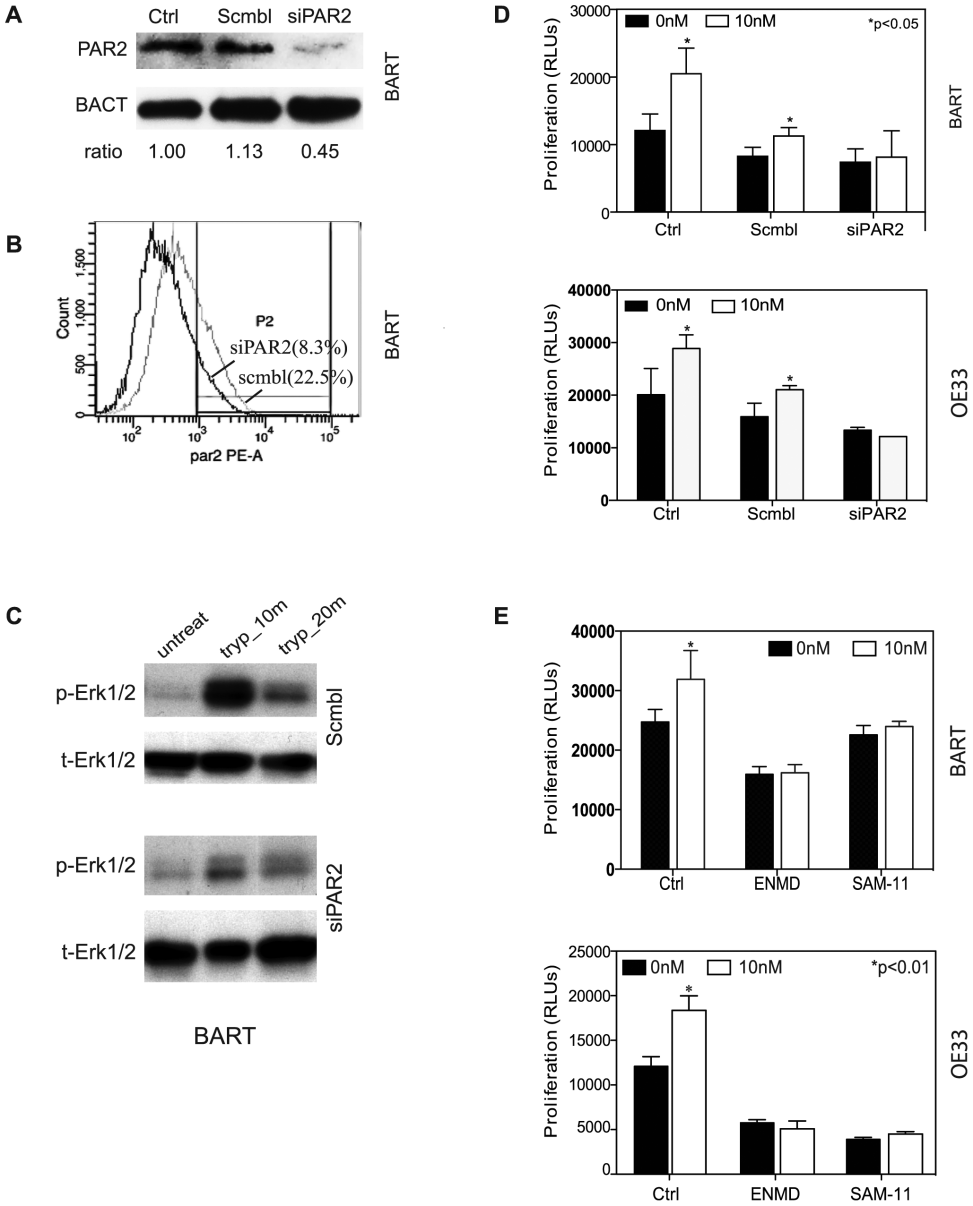
Effect of depletion or inactivation of PAR-2 on Erk1/2 phosphorylation and cell proliferation. (A) Western blot analysis using anti-PAR-2 antibody N-19 showed that the siRNA against PAR-2 caused a significant decrease (55%) in PAR-2 expression in BART cells compared with scrambled treatment. (B) Flow cytometry analysis further confirmed that the knockdown of PAR-2 led to a 60% reduction in PAR-2 surface expression in comparing with scrambled control cells. (C) siRNA against PAR-2 abrogated trypsin-induced Erk1/2 phosphorylation in BART cells. (D) Showing that siRNA knockdown PAR-2 inhibited trypsin-induced cell proliferation in both BART and OE33 cells. Ctrl: non-transfection; Scmbl: scramble control; siPAR2: siRNA PAR-2 knockdown. (E) Inhibition of PAR-2 activity by PAR-2 antagonist ENMD or anti-PAR-2 antibody SAM-11 abolished trypsin-induced proliferation.

PAR-2 is the main PAR member activated by trypsin-like serine proteases. To ascertain that PAR-2 is essential for trypsin induced signaling and cell proliferation, we used small interfering RNAs (siRNAs) to genetically deplete PAR-2 protein in cultured BART cells. By immunoblotting, an efficient knockdown of over 60% of PAR-2 protein expression was observed after 72 hours of transfection of siRNA against PAR-2 (figure 4A). Depletion of PAR-2 surface expression was observed by flow cytometry analysis using live cell staining for PE labeled anti-PAR-2 antibody (figure 4B). We then evaluated how siRNA-mediated knockdown of PAR-2 protein affected trypsin-induced phosphorylation of Erk1/2 in BART cells. As shown in figure 4C, trypsin induced Erk1/2 phosphorylation was observed in scramble siRNA control group, while this effect was virtually abolished in PAR-2 genetically depleted BART cells. To assess the functional importance of PAR-2 in mediating BART and EA cell proliferation, PAR-2 genetically depleted BART and OE33 cells along with their counterpart control scrambled siRNA transfected cells were subjected to ATP measurement. As shown in figure 4D, despite some off-target effect due to non-specific oligo transfection, the scrambled siRNA transfected cells retained a significantly augmented cell proliferation after 24 hrs incubation with 10 nM trypsin (p<0.01) in both cell lines. However, this trypsin-accelerated cell proliferation was abolished in PAR-2 siRNA treated cells.

Because siRNA is difficult to deliver *in vivo*, we then tested the effect of other forms of PAR-2 inactivation on cell proliferation. PAR-2 antagonist ENMD and anti-PAR-2 antibody SAM-11 were used to selectively inactivate PAR-2. Treating with ENMD (500 µM) for 2 hrs inhibited the proliferative response in both BART and OE33 cells to 10 nM trypsin (figure 4E). Treating BART and OE33 with anti-PAR-2 SAM-11 antibody (2 µg/ml) demonstrated very similar effect as small molecule PAR-2 inhibitor ENMD. Despite the PAR-2 antibody SAM-11 having less inhibitory effect on baseline proliferation in BART cells, it effectively blocked trypsin stimulation. Taken together, these data provided strong evidence that trypsin acts as growth factor in Barrett’s metaplasia and EA cells, and that the proliferation is induced by trypsin-PAR-2 activation of the MAPK signaling pathway.

### PAR-2 Inactivation Induces Apoptosis in EACs

**Figure 5:**
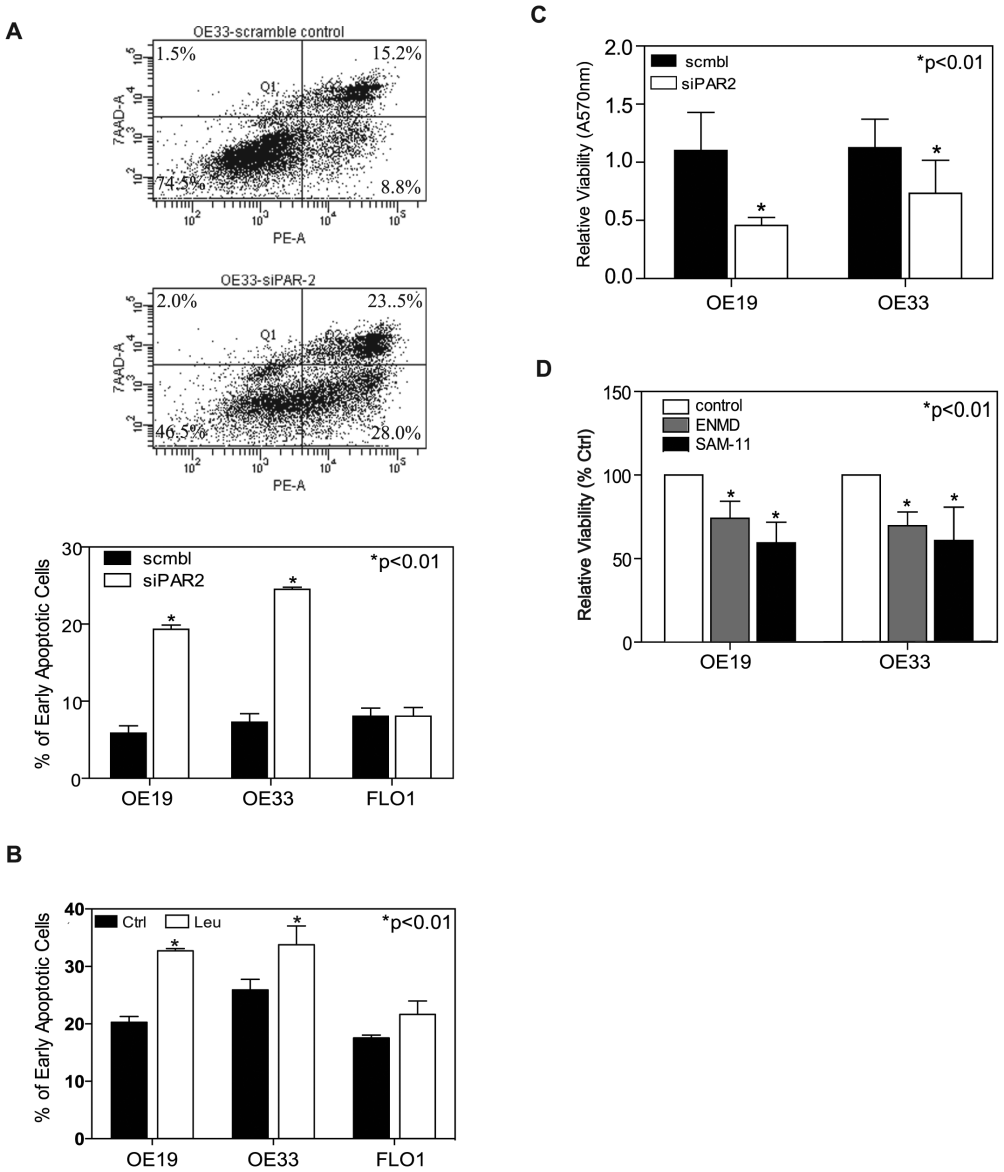
Effects of depletion and inactivation of PAR-2 on cell apoptosis. (A) Representative counter plot of Annexin V v.s. 7-ADD staining for apoptotic cells measured by flow cytometric analysis upon siRNA PAR-2 knockdown in OE33 cells; (B) Apoptosis is expressed as a percentage of early apoptotic cells (right lower quadrant) in total cell population for OE19, OE33 and FLO1. (C) Effect of siRNA PAR-2 knockdown on cell viability measured by MTT assay in OE19 and OE33 cells. (D) PAR-2 inactivation with its antagonist ENMD or anti-PAR-2 antibody SAM -11 decreased cell viability in comparing with untreated control cells in OE19 and OE33 cell.

In order to determine whether PAR-2 activation might also provide survival signaling in EAC, we used siRNA to knockdown PAR-2 expression in EAC followed by apoptosis analysis. In three separate experiments, the fraction of early apoptotic cells in OE19 increased from 5.85±1.05% (scrambled control) to 19.35±0.55% (PAR-2 siRNA knockdown, p<0.01). OE33 cells showed a similar trend from 7.30±1.10% (scrambled control) to 24.5±0.30% (PAR-2 siRNA knockdown, p<0.01). Interestingly, given the lower level of PAR-2 expression and trypsin secretion, PAR-2 siRNA knockdown in FLO1 cells did not alter its apoptotic rate (figure 5A). To confirm these results, we inhibited trypsin with the protease inhibitor leupeptin which also resulted in a significant increase in the fraction of apopotic cells in both OE33 and OE19, but not FLO1 (figure 5B).

We also used MTT assay to determine the effect of PAR-2 inactivation on cell viability. At 72 hrs after knockdown of PAR-2 expression, cell viability was decreased to 45.64±8.69% in OE19 cells and to 56.96±11.37% in OE33 cells compared to scrambled control cells (figure 5C). Furthermore, blocking PAR-2 activation by its antagonist ENMD (500 µM) and anti-PAR-2 antibody SAM-11 (2 µg/ml) also resulted in loss of cell viability: In OE19 cells, cell viability decreased to 74.17±10.09% (p<0.01) and to 59.44±12.41% (p<0.01) in treatment with ENMD and SAM-11 respectively; In OE33 cells, loss of viability was decreased to 69.69±8.22% (p<0.01) and 60.83±19.96% (p<0.01) respectively (figure 5D). These data suggested that PAR-2 activation plays an important role in the anti-apoptotic process in EACs.

### Autocrine Secretion of Trypsin in EACs Activates Proliferation and Survival Through PAR-2 Activation

**Figure 6:**
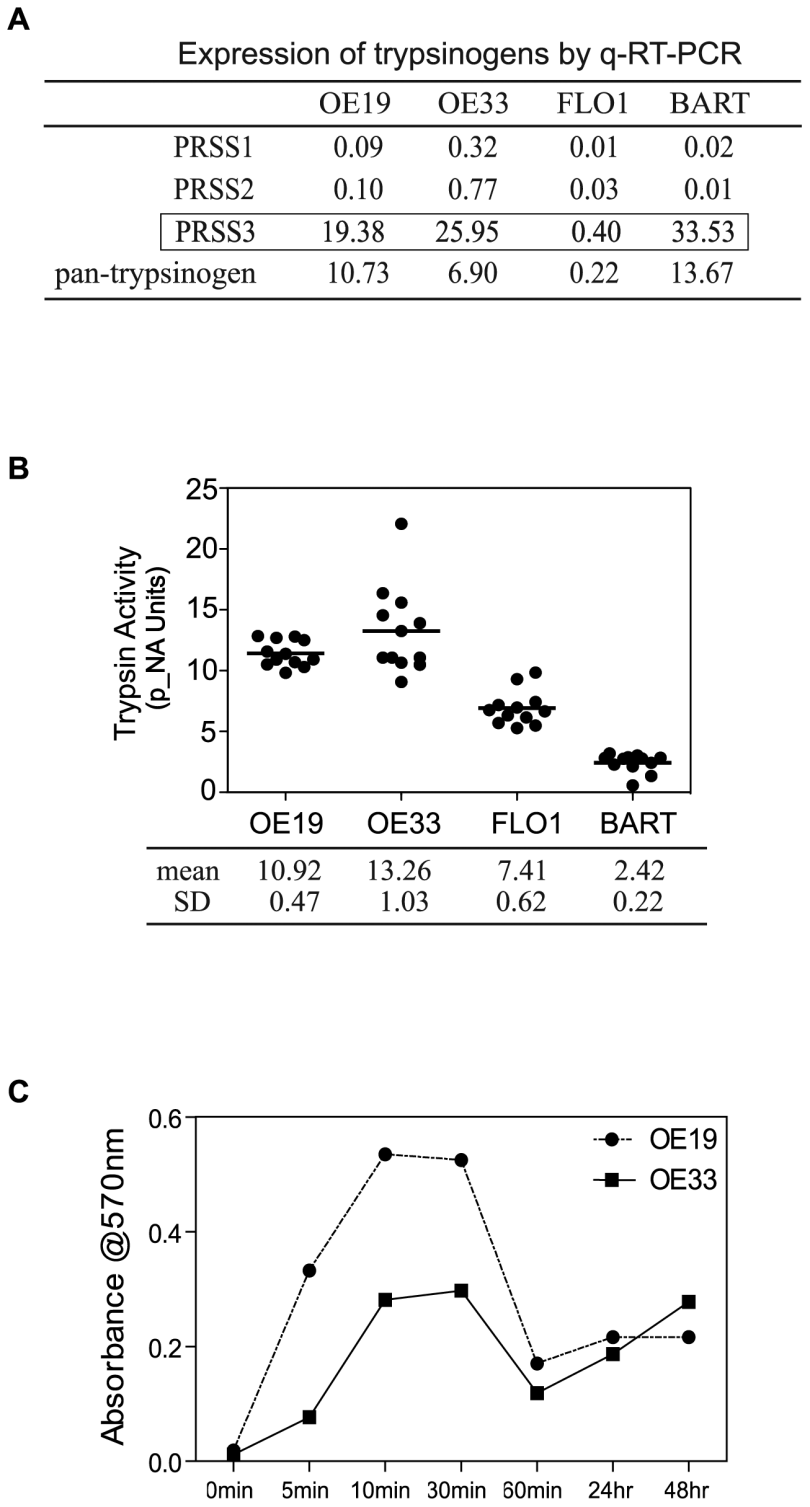
Profiling expression of trypsinogens and autocrine trypsin activity. (A) Quantitative RT-PCR analysis of RNA expression of trypsinogens in EA cells and BART cells. Table shows the preferential transcriptional expression of type 3 trypsinogen (PRSS3). (B) Trypsin enzymatic activities in cultured media were determined by BioVision Trypsin Activity Assay Kit. Trypsin activities were calculated and converted into *p*-NA units and are presented as mean±SD (*n* = 12), three experiments of quadruplicates tested. (C) Trypsin activities in cultured media from OE19 and OE33 were detected by BioVision Trypsin Activity Assay Kit over up to 48 hrs time courses, curves indicating the kinetic secretion of trypsin of both cells.

Human pancreatic cells synthesize and secrete three types of trypsinogen: the two major types are type 1 and type 2, with type 3 playing a minor role. Extra-pancreatic trypsinogens [namely tumor-associated trypsinogens (TATs)] were first identified by Koivune and colleagues, who purified and characterized TAT1 and TAT2 from cyst fluid of human mucinous ovarian tumors. Based on our observation of a blunted proliferation response of EA cells to exogenous trypsin, and the observation that PAR-2 inhibition reduced baseline proliferation and increased baseline apoptosis in EA cells, we speculated that they might secrete trypsin in an autocrine fashion. In this study, we examined the transcription profiles of trypsinogens in BART cells and EACs using quantitative real-time PCR. Pan-trypsinogen transcripts were detected in all cell lines, although weakly expressed in FLO1 cells (figure 6A). Quantitative real time PCR showed that type 3 typsinogen was preferentially transcribed in BART, OE19 and OE33 cell lines at a level of approximately 20–30 copy numbers per 1000 copy numbers of β_2_M, with only weak RNA expression in FLO1 (<1 copy number per 1000 copy numbers of β_2_M). Type 1 and type 2 trypsinogens were transcribed at comparatively lower levels in all cell lines (figure 6A). To confirm the q-RT-PCR finding, autosequencing was then applied to further characterize the specific type of human trypsignogen amplified. DNA sequence from reverse transcription PCR amplicons from BART cells and OE19, OE33 cells showed 100% sequence homology with trypsinongen 3 sequence (gene ID: PRSS3, NM_002771) by DNA sequence alignment software Clustal Omega (EMBL-EBI webside). DNA sequence mismatches with type 1 (gene ID: PRSS1, NM_002769) and type 2 (gene ID: PRSS2, NM_002770) trypsinogen sequences were observed (figure S1). This data demonstrates that BART, OE19 and OE33 cells preferentially express type 3 trypsinogen at the mRNA level.

### Trypsinogen is a Secreted Protein

Autocrine secretion of trypsin in cultured colon cancer cell line has been reported [12]. We thus set to examine the presence of secreted trypsin from cultured media of these cell lines. In conditioned media collected from cultured OE19 and OE33 cells trypsin activities were detected at a level equivalent to approx. of 10 nM (10.92±047 and 13.26±1.03 arbitrary units respectively). Trypsin activity in cultured conditioned medium from FLO1 cells was found at a lower, nearly undetectable level. Trypsin activity in the media of BART cells was below the detectable level (Figure 6B). EA cells dynamically secrete trypsin into culture media. The augmented trypsin levels appeared as early as 5 min and quickly reached peak concentration between 10–30 min, and thereafter gradually decreased to steady state for as long as 48 hrs in culture (Figure 6C). These data suggest that EA cells are able to express sufficient trypsin to initiate proliferation, and autocrine secretion of trypsin may sustain proliferation in esophageal adenocarcinoma.

### Inactivation of PAR-2 Confers Chemosensitivity to 5FU in EACs

**Figure 7:**
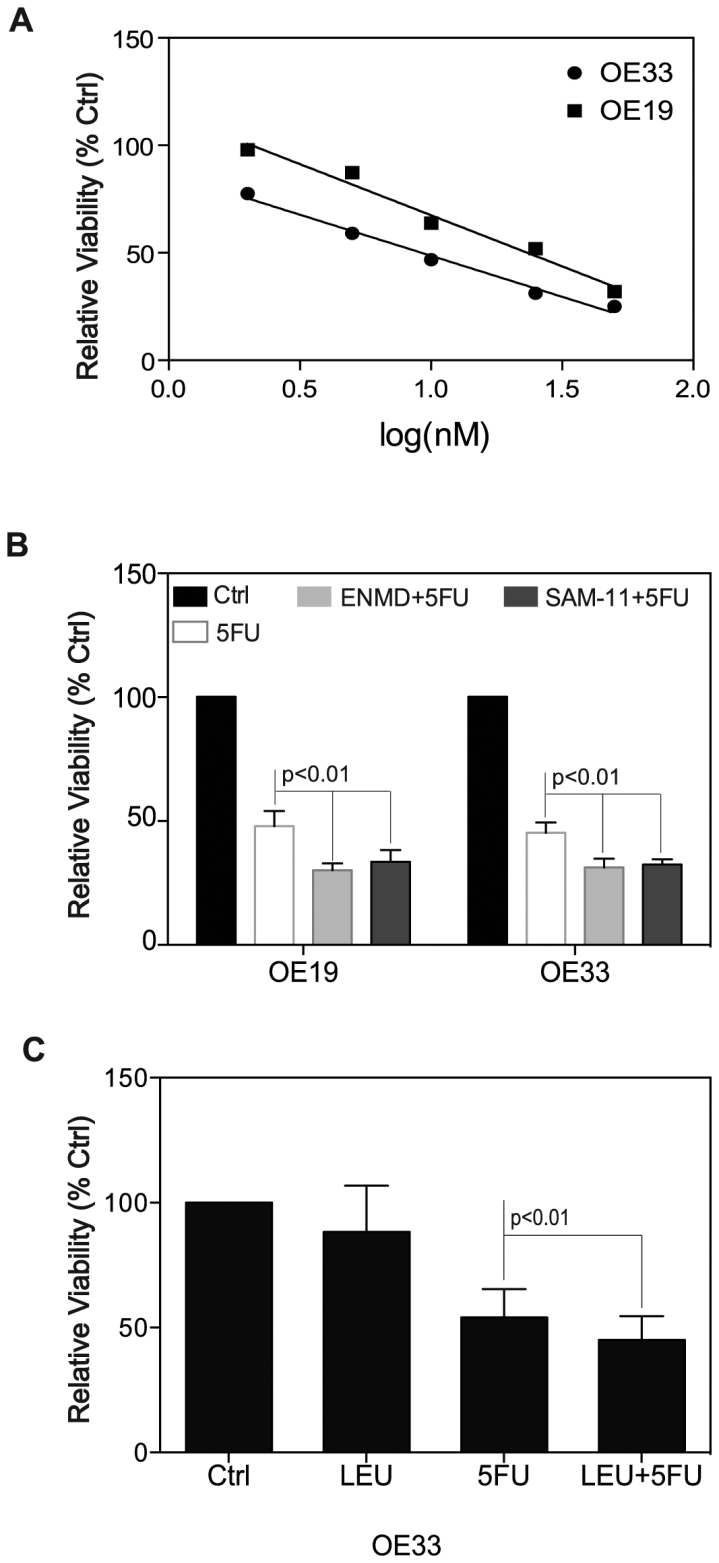
Inactivation of PAR-2 confers a synergetic effect on 5FU. (A) Dose-response curve of OE19 and OE33 cells to therapeutic agent 5FU. Cell viability measured by MTT assay. IC_50_ = 23.3 µM for OE19 cells and IC_50_ = 9.6 µM for OE33 cells. (B) In combination of blockade of PAR-2 activity by its antagonist ENMD (500 µM) or anti-PAR-2 antibody SAM-11 (2 µg/ml), 5FU (20 µM for OE19 and 10 µM for OE33) exerted higher cytotoxicity on OE19 and OE33 cells compared with 5FU alone (p<0.01). (C) Addition of 10 µg/ml leupeptin increases the effect of 5FU on OE33 cell viability.

Chemotherapeutic agents, such as 5FU have proven to have a limited impact on patients with esophageal adenocarcinoma due to chemoresistance. We speculated that inactivation of PAR-2 may resensitize EAC to 5FU via inhibiting cell proliferation and/or inducing apoptosis. 5FU treatment caused a dose-dependent cell death in both OE19 and OE33 cell lines with IC_50_ = 23.3 µM for OE19 cells and IC_50_ = 9.6 µM for OE33 cells (figure 7A). We thus selected 20 µM 5FU for OE19 and 10 µM 5FU for OE33 as our model treatment for the remainder of our experiments.

For the combinational treatment, both cell lines were pre-treated with 500 µM of ENMD or 2 µg/ml of anti-PAR-2 antibody SAM-11 for 2 hrs, followed by 5FU treatment at concentration of 20 µM for OE19 cells and 10 µM for OE33 cells for 72 hours. The combined regimen showed higher cytotoxicity in both cell lines than each drug alone (figure 7B). In OE19 cells, the survival rates of single treatment with 5FU compared to combinational treatment of ENMD+5FU or SAM-11+5FU were 45.2% v.s. 34.9% (p<0.01) and 45.2% v.s. 33.6% (p<0.01) respectively; In OE33 cells, the survival rates of single treatment with 5FU compared to combinational treatment of ENMD+5FU or SAM-11+5FU were 45.3% v.s. 31.4% (p<0.01) and 45.3% v.s. 32.4% (p<0.01) respectively. These data indicated that inhibition of PAR-2 activity rendered OE19 and OE33 cells more sensitive to chemotherapeutic agent 5FU.

To test whether trypsin inhibition could also sensitize EA cells to the chemotherapeutic agent 5FU, OE33 cells were pre-treated with trypsin inhibitor leupeptin (10 µg/ml) prior to exposure to 5FU treatment. As shown in figure 7C, a synergetic effect similar to PAR-2 inactivation (figure 7B) was observed. Leupeptin at 10 µg/ml alone has little inhibitory effect (88.37%, p>0.05), however, a further induced loss of cell viability was obtained at the presence of leupeptin in conjunction with 5FU treatment compared to single 5FU treatment (45.11% v.s. 54.08%, p<0.01). These data further support our hypothesis that inhibition of the trypsin/PAR-2 network could facilitate the effect of chemotherapeutic treatment in EA cells.

## Discussion

Our data show that PAR-2 and extra-pancreatic trypsinogen-3 (possible TAT-3) are both expressed in cell lines derived from human esophageal adenocarcinoma and Barrett’s esophagus, and exposing cells to trypsin accelerated cell proliferation *in vitro*. Further, we have shown that the adenocarcinoma cell lines studied here secrete trypsin into the culture media. Our data suggest that secreted trypsin acting on the PAR-2 receptor can function as an autocrine survival signal, and that targeting this axis can increase sensitivity to chemotherapy in esophageal adenocarcinoma cell lines. In addition, previous e*x vivo* study confirmed PAR-2 expression in normal and diseased human esophagus, where the receptors are vulnerable to trypsin exposure [15]. Taken together, this study demonstrated that trypsin activates PAR-2 in esophageal epithelial cells and plays a role in both benign and malignant pathological conditions.

The human pancreas secretes three types of trypsinogens 1, 2 and 3 [16]. In the late 1980s, Stenman and colleagues purified two isoenzyme forms of trypsinogen from cyst fluid of human mucinous ovarian tumors, and named them tumor-associated trypsin(ogen) TAT-1 and TAT-2 [17]. Both TAT-1 and TAT-2 have identical cDNAs and protein sequences and similar substrate specificities to their non-malignant counterparts, pancreatic trypsinogen-1 and -2 respectively, but differ in their susceptibility to inhibition by protease inhibitors. Other investigators have studied and reported TAT-1/TAT-2 expression in various tumors such as pancreatic, colon, gastric cancers including recently in intrahepatic cholangiocarcinoma cells [18], [19]. Later, Cottrell et al demonstrated trypsinogen-3 (namely mesotrypsinogen) or its splicing variant (namely trypsinogen IV) in tumor-derived epithelial cell lines as well as in normal human colonic mucosa. Trypsin IV has been found to act as an agonist for PAR-2 and PAR-4 but not for PAR-1 [20]. Recently Jiang et al reported (type 3 extra-pancreatic trypsinogen) PRSS3 expression in the metastatic pancreatic cancer PaTu8988s cell line, but not in the non-metastatic PaTu8988t cell line. Over-expression of PRSS3 has been shown to promote pancreatic cancer cell proliferation as well as invasion *in vitro*, and tumor progression and metastasis *in vivo* [13]. Supportive evidence was found in breast cancer where PRSS3/mesotrypsin is upregulated in malignant T4-2 cells as compared to their nonmalignant progenitors [14]. This study is the first report of PRSS3 expression in human pre-malignant and malignant esophageal conditions. Intriguingly, although trypsinogen (mainly PRSS3) transcripts were present in cultured BART cells, trypsin activity was not found in the cultured medium. It is not yet clear why there is lack of autocrine trypsin activity in BART cells. One possible explanation/assumption could be that malignant transformation activates the autocrine trypsin activity of EA cells, promoting cell proliferation and cell viability, while in premalignant Barrett’s esophagus, trypsin in reflux material is the stimulant for proliferation and survival signaling.

This study is the first report to demonstrate the preferential transcript expression of extra-pancreatic trypsinogens-3 (possible tumor-associated trypsin 3, TAT-3) in human esophageal adenocarcinoma cell lines as well as in the human Barrett’s esophagus BART cell line. Trypsinogen-3 (or mesotrypsinogen) is resistant to common trypsin inhibitors due to its unique ability of degradation of trypsin inhibitors, i.e. pancreatic secretary trypsin inhibitor SPINK1 [21]. In this study, using quantitative real-time PCR we systematically examined all three isotypes of trypsinogen transcript expression in human EA cell lines OE19, OE33, FLO1 as well as in BART cells. Our work revealed that extra-pancreatic trypsinogen-3, a possible TAT-3, is the most constitutively expressed isoenzyme in all above cell lines. Future work of *in vivo* examination of extrapancreatic trypsignogen-3 expression in human esophageal adenocarcinoma tissues will be crucial to confirm this finding.

Despite a long line of evidence showing PAR-2 expression and activation in diseased GI tract, such as reflux-associated esophagitis or gastroesophageal reflux disease (GERD), colitis, acute pancreatitis and gastric ulcer[22]–[25], there are no studies to date whether PAR-2 plays a role in Barrett’s esophagus. Barrett’s esophagus (BE) is a premalignant condition that predisposes to the development of esophageal adenocarcinoma. Progressively increased cell proliferation has been proven as one of the cellular events during malignant transformation. Our previous work has shown that components of gastro-duodenal reflux such as the bile salt GCDA was capable of stimulating cell proliferation in BART cells via ERK phosphorylation. We speculated that reflux of protease trypsin may also play a part in malignant transformation of BE. The proliferative effect of trypsin/PAR-2 signaling in BART cell was observed in this work. Given our findings, it is plausible to link PAR-2 activation with BE malignant transformation due to the risk of provoking uncontrolled cell growth.

Likewise, mounting evidence suggested that PAR-2 activation induces phosphorylation of ERK MAPK pathway, resulting in accelerated cell proliferation in various epithelial cancers. *In vitro* studies showed that activation of PAR-2 enhances the growth of two gastric cancer cell lines AGS and MKN28 via EGFR trans-activation. Autocrine trypsin production and PAR-2 activation significantly increasing cell proliferation along with enhancement of MAP kinase was observed in pancreatic and colonic cancers [5], [26]. Treating human colon cancer cell HT-29 with 0.1–1.0 nM trypsin [27] elicited a highly significant increase of cell number *in vitro*. *In vivo* subcutaneous xenografted tumors showed significantly enhanced growth after treated with PAR-2 agonist in pancreatic cancer [5]. Using the established esophageal adenocarcinoma cell lines in this study, we also demonstrated proliferative response to trypsin stimulation in these cell lines, with more effect in OE19 and OE33 cells where trypsin was more abundantly expressed. Our data suggest that EACs, like other GI tract tumors, synthesize and secrete trypsin into the microenvironment, creating an autocrine loop that consequently cleaves cell surface PAR-2, resulting in proliferation.

Thus far, previous studies have revealed the role of PAR-2 in tumor pathology with focus on its effect of inciting cell proliferation and migration [11]. Yada et al. reported the utilization of anti-PAR-2 neutralizing antibody for suppressing PAR-2 induced cell proliferation, however the effect of targeting PAR-2 therapeutically remains unclear. This study is the first report that PAR-2 inhibition not only blocked cell proliferation in EACs and BART cells, also induced apoptosis in EACs. We further explored the strategy of targeting PAR-2 in combination with current chemotherapy. By inactivating PAR-2 either with its antagonist ENMD or PAR-2 antibody SAM-11 or siRNA depletion of PAR-2, we were able to demonstrate that inhibition of PAR-2 activation sensitized EACs to the current chemotherapeutical agent 5FU and conferred a synergistic cell killing effect *in vitro*. We reason that potent PAR-2 antagonists/inhibitors may provide chemotherapeutic sensitization in cancer patients by blocking trypsin mediated survival signaling and sensitizing cells to other apoptosis inducing compounds such as chemotheraputic agents. Nafamostat mesilate and gabexate mesilate are synthetic low molecular weight broad-specificity protease inhibitors. Recent studies have shown that nafamostat mesilate potentiated gemcitabine anti-tumor effect by inhibiting gemcitabine induced NF-kB activation in pancreatic cancer [28], [29]. Likewise, the anti-proliferation/anti-invasion effect of gabexate mesilate has been reported in pancreatic cancer cells attributed to its inhibition of trypsin activity [30]. Although it is uncertain whether nafamostat mesilate functions via suppressing PAR-2 activation [31], [32], it has shown promise in combination with gemcitabine in a phase I/II clinical trial for pancreatic cancer treatment [33], [34]–an encouraging example of how employing trypsin/PAR-2 activation blockade agents in combination with current chemotherapy might provide synergy in esophageal cancer treatment.

In summary, we have shown that PAR-2 was constitutively expressed in cultured EACs and BART cells and trypsin exposure resulted in increased cell proliferation via trans-activation of ERK MAPK pathway. We have also demonstrated that EAC’s secrete trypsin, which appears to act via an autocrine loop to enhance cell survival. More importantly, we provided clear evidence that targeting PAR-2 increases apoptosis in EACs and consequently confers chemo-sensitization of EACs. Our data suggest a role for the development of potent PAR-2 suppressors, which may represent an attractive novel adjunct to current cytotoxic chemotherapy.

## Supporting Information

Figure S1 **DNA sequences alignments.** DNA sequences generated from PCR amplification with pan-trypsingoen primers from OE33 cell were aligned with gene sequences of all three types of trypsinogenes. Partial sequences of the PCR amplicon shown. Sequences for trypsinogen type1 (PRSS1, NM_002769); trypsinogen type2 (PRSS2, NM_002770) and trypsinogen type3 (PRSS3, NM_002771) were retrieved from NCBI gene bank. Mismatch bases were shown in red.
(TIFF)
