# Supplementary figures and images for: Autocrine Extra-Pancreatic Trypsin 3 Secretion Promotes Cell Proliferation and Survival in Esophageal Adenocarcinoma
Source: PLoS One. 2013 Oct 11;8(10):e76667. doi: 10.1371/journal.pone.0076667 (PMC3795734; doi:10.1371/journal.pone.0076667)

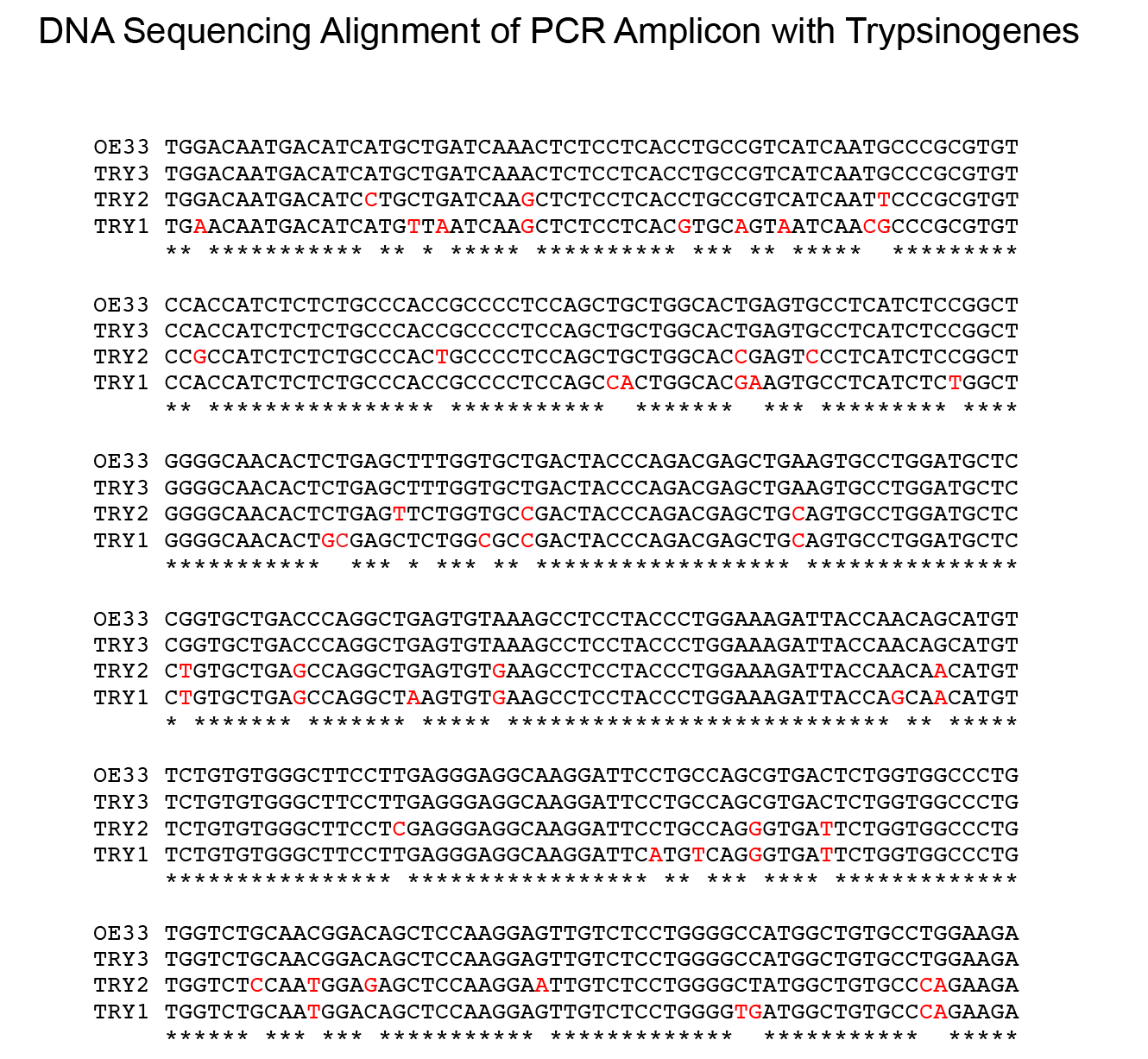

Supplement: Figure S1 — DNA sequences alignments. DNA sequences generated from PCR amplification with pan-trypsingoen primers from OE33 cell were aligned with gene sequences of all three types of trypsinogenes. Partial sequences of the PCR amplicon shown. Sequences for trypsinogen type1 (PRSS1, NM_002769); trypsinogen type2 (PRSS2, NM_002770) and trypsinogen type3 (PRSS3, NM_002771) were retrieved from NCBI gene bank. Mismatch bases were shown in red. (TIFF) [file pone.0076667.s001.tiff]
